# Fertility preferences among couples in Nigeria: a cross sectional study

**DOI:** 10.1186/s12978-020-00940-9

**Published:** 2020-06-11

**Authors:** Emmanuel Kolawole Odusina, Titilayo Ayotunde, Michael Kunnuji, Dorothy N. Ononokpono, Ghose Bishwajit, Sanni Yaya

**Affiliations:** 1 Department of Demography and Social Statistics, Federal University, Oye-Ekiti, Nigeria; 2 Department of Demography and Social Statistics, Obafemi Awolowo University, Ile-Ife, Nigeria; 3 Department of Sociology, University of Lagos, Lagos, Nigeria; 4 Department of Sociology and Anthropology, Faculty of Social Sciences, University of Uyo, Uyo, Nigeria; 5 School of International Development and Global Studies, University of Ottawa, Ottawa, Canada; 6 The George Institute for Global Health, The University of Oxford, Oxford, UK

**Keywords:** Fertility desire, Type of marriage, Couple, Nigeria, Demographic and health survey, Cross-sectional study; Global Health

## Abstract

**Background:**

The persistently high and stalled total fertility in Sub-Saharan Africa, including in Nigeria, calls for new efforts towards fertility reduction. Most efforts on fertility desire in sub-Saharan Africa have focused either on individual men or women with little focus on couples as a unit of analysis. Moreover, the influences of different types of marriages in which couples reproduce have not been adequately explored. Therefore, this study examined fertility desires among couples in Nigeria.

**Methods:**

This paper used data from the Nigeria Demographic and Health Survey (NDHS) of 2018 to assess fertility desire by marriage type among couples in Nigeria. In addition, the association between fertility desire and disparity in couples’ educational attainment, place of residence, region, religion, occupation, wealth status, children ever born and contraceptive use were considered. The participants consisted of 6813 couples aged between 15–49 years. Couples’ characteristics were reported using frequency and percentage distribution tables. Descriptive and logistic regression analyses were conducted.

**Results:**

Overall, the study revealed that 73.8% of couples were in monogamous relationships while 26.2% were in polygynous relationships. The mean ideal number of children for men and women were 7.2 and 6.1, respectively. Also, 49.3% of the couples reported husbands desired more children, 43.9% claimed wives desired more children, while 6.8% indicated equal number of desired children among wives and husbands. The results of binary logistic regression showed that couples in polygynous relationships were 4.3 times as likely to desire more children, compared to couples in monogamous relationships (OR = 4.3; 95% CI: 3.5, 5.3). Couples in polygynous relationships wanted as many as four times the number of children desired by couples in monogamous relationships. Fertility desire was significantly higher among couples who indicated the following: either was using contraceptives (OR = 2.3; 95% CI: 1.6–3.4), both were not using contraceptives (OR = 2.8; 95% CI: 1.9, 4.1), lived in North East (OR = 2.0; 95% CI: 1.5, 2.6) and North West (OR = 1.7; 95% CI: 1.3, 2.3), both were not working (OR = 1.33, 95% CI; 1.1, 1.6) and were adherents of Islam (OR = 1.8; 95% CI; 1.5, 2.4).

**Conclusion:**

These findings reflect the role of region, use of contraceptives, work status and religion in the fertility desire of couples. Implementing programmes and policies on sexual education and reproductive rights of couples and individuals may reduce high fertility desire and its adverse consequences, such as child and maternal morbidity and mortality in Nigeria.

## Plain English summary

The total fertility rate in West African countries remains high despite the development and implementation of several interventions. The literature identifies significant factors relating to fertility desires and subsequent population growth at individual, household and community levels. Nevertheless, the high and stalled total fertility rate of Nigeria at 5.3 calls for new approaches to affect fertility. Marital fertility constitutes a substantial part of total fertility in Nigeria. Previous studies mostly focused on the fertility desires of men and women. Motivated by an emerging interest in fertility intentions, the study examined fertility preferences among monogamous and polygynous couples in Nigeria. Data for the study were retrieved from Nigeria Demographic and Health Survey (NDHS) of 2018. A total sample of 6813 couples between 15 and 49 years of age was used in the study. Analyses were done using frequency and percentage distribution tables and a logistic regression model. Couples in polygynous relationships wanted about four times the number of children desired by couples in monogamous relationships. There were disparities in socio-demographic characteristics of couples and contraceptive use by type of marriage. Factors influencing the desire for more children among couples were work status, region, religion and contraceptive use. Existing interventions should take these factors into consideration.

## Background

Achievement of Sustainable Development Goals (SDGs), especially goals one (end all forms of poverty), three (ensure healthy lives and promote well-being for all at all ages), four (ensure inclusive and equitable quality education and promote life-long learning opportunities for all) and five (achieve gender equality and empower all women and girls) might be a mirage without conscious efforts on the part of many developing countries to reduce population growth [1–3]. Rapid population growth due to a high fertility rate and fertility desire may engender strife, poverty, unemployment, competition for scarce resources, etc. [3]. A persistently high fertility rate and fertility desire will make developing countries poorer as a result of its effects on social and health care development [2]. A high fertility rate leads to a large population and this may subsequently lead to stiff competition for scarce resources that could instead be used for further productivity and income generation [2].

Generally, many developing countries of the world have witnessed development in their demographic transition. This has shown in the tremendous reduction in fertility and mortality rates over the last few decades [4–6]. However, total fertility rates in the West African sub-region have remained high [7, 8]. It is 5.3 per woman in Nigeria, 6.0 in Burkina Faso, 6.6 in Mali and 7.6 in Niger Republic [8]. In a bid to have a sustainable fertility reduction strategy and prevent unwanted, untimely and high-risk pregnancies, many countries have tried several population policies, including embracing family planning as one of the several methods of controlling population growth rate. Nigeria’s fertility rate, for instance, remained high and stalled after 28 years of different population policies [9–11]. Over the years, the country’s fertility rate, which was 6.6 in the 1960s, moved upward to 7.0 in 1975 [10] and later dropped to slightly less than 6 births per woman in the recent decades [8, 12].

Introduced in 1988, the National Policy on Population for Development, Unity, Progress and Self-Reliance set the following targets for one of its goals: ‘To achieve lower population growth rates, through reduction of birth rates by voluntary fertility regulation methods that are compatible with the attainment of economic and social goals of the nation.' The policy focused on individuals and couples in monogamous and polygynous families. This focus was necessary because marital fertility constituted a substantial part of the fertility in Nigeria. Moreover, this was not unconnected with the fertility desire of the monogamous and polygynous families. The total fertility rate was 6.0 in 1990. It was 5.7 in 2003, about fifteen years after the policy on population was introduced. The policy was revised and replaced in 2005 with the National Policy on Population for Sustainable Development, which set the following targets for one of its goals: ‘Progress towards a complete demographic transition to a reasonable growth in birth rates and a low death rate.' Three years later (in 2008), the total fertility rate remained 5.7. Eight years later (in 2013), the total fertility rate reduced to 5.5, a rate that was sustained for several years of population policies. The 2018 NDHS released in 2019 put the figure at 5.3 [7].

This has provoked a series of studies on the significant predictors of fertility in the country. Many empirical biosocial studies have revealed significant individual, household, kinship and community determinants of fertility rates in sub-Saharan Africa and Nigeria, in particular [10]. Importantly highlighted among these determinants of fertility rate and subsequent high population growth is fertility desire [13, 14]. However, as important as fertility desire is in the determination of fertility rate cum population growth, substantial scholarly enquiries have not been accorded to this germane research focus. The belief that more has to be done to explore or highlight these significant predictors of population growth path motivates the present study. The high total fertility and persistent population increase - despite different population policies - call for new approaches and efforts to influence the level of fertility in Nigeria. This is because properly planned population growth may foster adequate welfare of citizens, provision of requisite infrastructural development and reduction in the level of poverty in the country. Before making any efforts in these directions, one of the pertinent questions to ask is ‘How many more kids does a man in a polygynous relationship want than his wife compared to his counterpart in a monogamous relationship?’ or ‘Does a man in a polygynous relationship have higher odds of wanting more children than his wife compared to a man in a monogamous relationship?’ Adequate knowledge of fertility desire by marriage types (monogamous and polygynous families) would aid understanding and deepen knowledge on fertility intention.

Research has shown that most births are within marital unions [7, 8]. Consequently, a significant reduction in marital fertility will invariably reduce total fertility in Nigeria [8]. However, with regards to fertility, most studies have focused either on individual men or women [15–20] with few studies on couples [10, 21, 22]. In addition, type of marriage has not been adequately explored as a possible factor in fertility desire and consequently on the reproductive health of couples. Information on couples’ fertility desire, which mostly occurs in a marital setting, may help to explain and provide information necessary to influence the tide of fertility rate in Nigeria. It is therefore imperative to examine the relationship between fertility desire and marriage types among couples in Nigeria.

Nigeria, a country endowed with natural resources, can enjoy demographic dividend. This includes couples with other achievements, decline in mortality rates, increase in productivity of the working population, quality of education, adequate nutrition and access to sexual and reproductive health and rights. Also, there have been significant reductions in fertility and there are still conscious efforts being made. The reduction in fertility rate in Nigeria may reduce population growth and pave way for socio-economic development in the long run. Demographic dividend may facilitate economic growth and also enhance the possibility of quality education, good health and adequate nutrition. Change in population age structure due to a higher proportion of working people in the total population will increase the potential for economic growth and development.

This study is underpinned by the polygyny-fertility hypothesis; this hypothesis associates reduced fertility with polygyny. Theoretically, studies on the polygyny-fertility hypothesis showed mixed results. Many of the studies explained that fertility was lower in polygynous marriages compared to monogamous marriages and that inconsistency occurred due to differences in fertility by wife-order, number and aggregation of fertility among women in a polygynous marriage [23, 24]. On the contrary, using the polygyne hypothesis, Mulder's study on marital status and reproductive performance in Kipsigis women identified no association between marriage order or the number of wives in a polygynous marriage and reproductive performance [24]. Nigeria was noted for polygynous marriage, but the association between polygyny and fertility in Nigeria had not been explored adequately [25]; the dearth of evidence on the fertility-polygyny relationship may hinder efforts and intervention programmes especially in Nigeria. This stresses the need for more research on couples and their fertility desires. Therefore, this study hypothesized that a man in a polygynous relationship has higher odds of wanting more children than his wife compared to his counterpart in a monogamous relationship.

Given the emerging interest in the study of fertility intention among couples in developing countries, this study examined the extent to which couples in marriage types desire for children and the factors associated with it in Nigeria. Previous attempts at explaining fertility desire focused mainly on women or men [15, 16, 19].

## Methods

### Data extraction

Data for this study were derived from the 2018 NDHS couples’ recode dataset. It was a cross-sectional, comparable and nationally representative survey that employed a three-stage sampling technique. Taking into consideration the probability proportional to enumeration area size, 1400 Enumeration Areas (EAs) were selected. In all selected EAs, a household listing was done and the lists of households were used as a sampling frame. Nigeria was divided into clusters based on the 2006 Enumeration Areas (EAs) census frame and demarcation. In each cluster, 30 households were selected randomly to arrive at a representative sample of 42,000 households. Women in the 15–49 age range who were permanent residents or visitors in the household the night before the survey were considered eligible to participate in the interview.

The survey involved three different questionnaires. These were the household questionnaire, women’s questionnaire and men’s questionnaire. Also, a sub-sample of half of the men in the 15–49 age range in selected households was also interviewed [8]. Objective and self-reported information was collected on indicators of family planning, reproductive health, fertility, child and maternal health, nutrition and mortality. The 2018 NDHS dataset is available at http://dhsprogram.com/data/available-datasets.cfm. The records of women and men interviewed were matched to derive the couples’ dataset set. This study was based on ‘couple’ as a unit of analysis; hence, couples’ recode dataset was used for the study. Responses to questions on fertility desire and other relevant variables were included in the analysis. Non-response and those who indicated that they 'did not know' to questions were excluded to arrive at the weighted sample size of 6813.

### Study variables and their measurement

Fertility desire was the outcome or dependent variable. It had three categories: ‘equal desire,' ‘husbands desire more’ and ‘wives desire more.' It was further reduced to two categories (equal desire {reference group coded ‘0’} and husbands desire more {coded ‘1’}. The third category, ‘wives desire more,’ was excluded in the course of data analysis (Table 3) because it had a few cases - 464 (6.8%) of the total sample size. The study had several independent variables: marriage type, disparity in couple’s educational attainment, region, place of residence, religion, wealth status, occupation, children ever born and contraceptive use. The study benefited from previous studies while considering variables to be included [10, 21, 22]. Fertility desires of husbands as reported by wives were used for analysis because a fertility report of the husbands was not available. In addition, reports of husbands on children ever born were used due to the polygynous nature of many families and the fact that a woman might report her own children ever born rather than that of the family. Table 1 provides the definition and coding of variables as used in the study.

**Table 1:** Variables used for analysis

**Study Variables**	**Operational Definitions and Coding**
Fertility desire	0 = Equal desire, 1 = Husbands desire more and 2 = Wives desire more
Type of marriage	1 = Monogamy and 2 = Polygyny
Region	According to respective regions of respondents as provided by NDHS, 1 = North Central, 2 = North East, 3 = North West, 4 = South South, 5 = South East and 6 = South West
Age difference	Self-reported ages of couples coded as 1 = same age (less than 3 years younger or older), 2 = husbands older (husbands more than 3 years older) and 3 = wives older (wives more than 3 years older)
Difference in Education	Self-reported number of years spent in school by couples coded as 1 = same education (for couples who spent the same number of years in school), 2 = husbands more educated (husbands spent more years in schools compared to wives) and 3 = wives more educated (wives spent more years in schools compared to husbands)
Wealth Index	According to household index, wealth is classified into 5 categories by NDHS, 1 = Poorest, 2 = Poorer, 3 = Middle, 4 = Richer and 5 = Richest
Residence	According to NDHS classification, 1 = urban and 2 = Rural
Religion	1 = Christianity, 2 = Islam and 3 = Traditional religionists and others
Work Status of Couples	1 = Both are working and 0 = Others (it involved couples who indicated that either were working or that both were not working)
Children Ever Born	1 = 0, 2 = 1–2, 3 = 3–4 and 4 = 5+
Use of Contraceptive	1 = Using and 2 = Not using

### Statistical analysis

Analyses of data were carried out at three levels – univariate, bivariate and multivariate with the use of Stata software. Frequencies and percentages were used at the univariate level to explain the socio-demographic and other characteristics of respondents while Chi- square tests were carried out at the bivariate level to consider the associations of fertility desire with socio-demographic and other variables. In addition, a correlation test was carried out to discover multicollinearity among independent variables. The results revealed the multicollinearity assumption was not violated. The tolerance value was not less than 0.10 (tolerance value of less than 0.1 is an indication of high correlations with other variables in the model) and the correlation between independent variables was less than 0.7 [26]. A binary logistic regression model was fitted at the multivariate level of analysis because the categories of the outcome variable were reduced from three to two. This study referred to and benefited from similar previous studies [10, 21, 22]. The model was adjusted for contraceptive use, differences in age, work status, wealth index, religion and number of children ever born to determine their association with fertility desire.

## Results

### Sample characteristics

Table 2 presents the socio-demographic and other characteristics of respondents. A majority of the couples belonged to a monogamous marriage type (73.8%), were working (69.3%), lived in rural areas (58.9%), were adherents of the Islamic religion (58.9%), were not using contraceptives (73.4%) and reported husbands were older (84.0%). Generally, the mean ideal number of children for men and women were 7.2 and 6.1, respectively. Almost half of the couples (49.3%) reported that husbands desired more children. The equal desired and more desired on the part of wives compared to husbands were 43.9% and 6.8%, respectively. About a third of the respondents were from North West (33.1%), 12.8% were from North Central, 17.9% from North East and 16.1% were from South West. Frequency distributions according to number of years spent in schools revealed that couples with about the same level of education had the highest percentage (46.2%). In consort, 39.2% of the husbands were more educated than their wives, with 14.6% of wives more educated than their husbands.

**Table 2:** Background and other selected characteristics and fertility desire: Univariate and bivariate analyses

**Background Characteristics**	Number	Percentage	**Fertility Desire**		**Chi-squared test**	
			Equal Desire	Husbands Desire More	**χ**^**2**^	***P*-Value**
**Marriage Type**						
Monogamy	5028	73.8	2734 (59.2)	1888 (40.9)		
Polygyny	1785	26.2	254 (14.7)	1473 (85.3)	971.4	*p* < 0.0000
**Region**						
North Central	872	12.8	447 (54.4)	375 (45.6)		
North East	1217	17.9	336 (28.7)	835 (71.3)		
North West	2257	33.1	585 (27.5)	1542 (72.5)	1076.7	*p* < 0.0000
South East	767	11.3	518 (75.8)	166 (24.2)		
South South	604	8.9	421 (77.3)	123 (22.7)		
South West	1097	16.1	682 (68.0)	321 (32.0)		
**Age**						
Same Age	1040	15.3	551 (57.6)	405 (42.4)		
Husbands Older	5723	84.0	2401 (44.9)	2944 (55.1)	66.9	*p* < 0.0000
Wives Older	51	0.7	37 (75.9)	12 (24.1)		
**Difference in Education**						
Same Education	3145	46.2	1292 (43.9)	1650 (56.1)		
Husbands more educated	2673	39.2	1174 (47.0)	1324 (53.0)	50.0	*p* < 0.0000
Wives more educated	996	14.6	522 (57.5)	386 (42.5)		
**Work Status**						
Both are working	4718	69.3	2278 (52.2)	2088 (47.8)		
Other	2095	30.7	710 (35.8)	1273 (64.2)	142.8	*p* < 0.0000
**Wealth Index**						
Poorest	1343	19.7	411 (31.7)	885 (68.3)		
Poorer	1368	20.1	429 (32.9)	877 (67.2)	563.2	*p* < 0.0000
Middle	1347	19.8	550 (43.6)	711 (56.4)		
Richer	1289	18.9	674 (57.0)	508 (43.0)		
Richest	1467	21.5	924 (70.9)	380 (29.1)		
**Residence**						
Urban	2801	41.1	1523 (60.0)	1014 (40.0)		
Rural	4012	58.9	1465 (38.5)	2346 (61.5)	277.1	*p* < 0.0000
**Religion**						
Christianity	2793	40.1	1802 (72.3)	691 (27.7)		
Islam	4012	58.9	1184 (30.8)	2663 (69.2)	1020.2	*p* < 0.0000
Traditional/Others	08	0.1	02 (24.8)	06 (75.2)		
**Children Ever Born**						
0	395	5.8	168 (44.9)	206 (55.1)		
1–2	2020	29.6	1009 (54.9)	831 (45.1)	117.8	*p* < 0.0000
3–4	1873	27.5	886 (50.7)	862 (49.3)		
5+	2526	37.1	926 (38.8)	1463 (61.2)		
**Contraceptive Use**						
Both are Using	330	4.8	241 (82.3)	52 (17.7)		
Both are not Using	4997	73.4	1906 (40.4)	2809 (59.6)	352.4	*p* < 0.0000
Either is Using	1486	21.8	841 (62.7)	499 (37.3)		
**Fertility Desire**						
Equal Desire	2988	43.9				
Husbands desire more	3361	49.3				
Wives desire more	464	6.8				

The percentage distribution of the couples by wealth status revealed that 39.8% belonged to the poor category, 19.8% belonged to the middle class category, while 40.4% belonged to the rich category. Fertility desires by couples’ characteristics were also examined. As also revealed in Table 2, all the socio-demographic and other selected variables (marriage type, age, education, wealth index, residence, children ever born and contraceptive use) were statistically significantly associated with fertility desire. 

### Multivariate analysis of determinants of couples’ fertility desire

This section presents the results of multivariate analysis (Table 3). The relationship between fertility desire and selected explanatory variables was examined using the odds ratios. The first category (equal desire) was coded as '0' while the second category (husbands desired more) was coded as '1.' A binary logistic regression model was fitted to examine the influence of selected independent variables on couples’ fertility desire.

**Table 3:** Multivariate Analysis of Selected Characteristics of Couples by Fertility Desire (0 = Equal desire and 1 = Husbands desire more)

**Variables**	OR	95% CI
**Marriage Type**		
Monogamy (RC)	1.00	
Polygyny	4.3^***^	3.5–5.3
**Contraceptive Use**		
Both are Using (RC)	1.00	
Both are not Using	2.8^***^	1.9–4.1
Either is Using	2.3^***^	1.6–3.4
**Region**		
North Central (RC)	1.00	
North East	2.0^***^	1.5–2.6
North West	1.7^***^	1.3–2.3
South East	0.8	0.6–1.1
South South	0.8	0.5–1.0
South West	1.1	0.8–1.4
**Age**		
Same Age (RC)	1.00	
Husbands Older	1.0	0.4–1.8
Wives Older	0.5	0.2–1.1
**Difference in Education**		
Same Education (RC)	1.00	
Husbands more educated	1.0	0.8–1.2
Wives more educated	1.1	0.9–1.4
**Work Status**		
Both are working (RC)	1.00	
Others	1.33***	1.1–1.6
**Wealth Index**		
Poorest (RC)	1.00	
Poorer	1.2	0.9–1.5
Middle	1.1	0.9–1.4
Richer	1.1	0.8–1.4
Richest	0.8	0.6–1.1
**Residence**		
Urban (RC)	1.00	
Rural	1.2	0.9–1.4
**Religion**		
Christianity (RC)	1.8^***^	
Islam		1.5–2.4
**Children Ever Born**		
0 (RC)	1.00	
1–2	1.1	0.8–1.4
3–4	1.3	1.0–1.7
5+	1.2	0.9–1.6

*RC* Reference category, *OR* odds ratio, ***p<0.001

The model revealed the adjusted odds ratios of the selected independent variables on fertility desire. This model revealed a positive, statistically significant, higher odds ratio for more desire for children by husbands (OR = 4.3; 95%CI: 3.5, 5.3; *P* < 0.001) in a polygynous marriage type relative to monogamy. The model also revealed positive, statistically significant odds ratio for more desire for children by husbands among couples who were not using contraceptive (OR = 2.8; 95%CI: 1.9, 4.1; *P* < 0.001), either was using contraceptive (OR = 2.3; 95%CI: 1.6, 3.4; *P* < 0.001) and among couples who were not both working (OR = 1.33; 95%CI: 1.1, 1.6; *P* < 0.001). In addition, this study found statistically significant odds ratio for more desire for children by husbands among the couples in North East (OR = 2.0; 95%CI: 1.5, 2.6; *P* < 0.001), North West (OR = 1.7; 95%CI: 1.3, 2.3; *P* < 0.001) and the couples who were adherents of Islam (OR = 1.87; 95%CI: 0.5, 0.9; *P* < 0.01).

RC = Reference category; OR = odds ratio; ^***^p < 0.001.

## Discussion

### Main findings

Overall, more couples reported a monogamous type of marriage compared to a polygynous type. This finding contrasted the study carried out by Turnwait and Alfred (2018) which indicated that Nigeria was noted for polygyny [27]. There were disparities in couples’ desire for children. Less than half of the couples had equal desire for children. Fertility desires of husbands were more than that of their wives. This was in line with studies elsewhere [21, 22]. In addition, among the couples in the sample population, fertility desires of husbands were more in a polygynous marriage compared to fertility desires of husbands in a monogamous marriage. This finding is supported by a study conducted by Tertilt [28, 29]. The finding on the association between religion and fertility desires attests to the fact that Islamic religion and customary law encourage a polygynous marriage, which in turn leads to high fertility [30, 31]. Islam permits a man to marry four or more wives and this explains the desire for more children found among husbands affiliated to Islam. The high fertility desire of husbands over that of wives showed the pronatalist tendency of men [21] in Nigeria. This tendency might be related to the roles and traditional expectations men play in Nigeria as the heads and breadwinners of most families, as well as being the older partners in most cases. Men were also expected to take the leading role in decision-making even including decisions primarly affecting their wives. The roles were supported by cultures and religions in the country. This realisation may have led to lower fertility desire on the part of wives compared to their husbands. The different fertility goals of husbands and wives were supported by other studies [21, 22, 32]. However, higher desire for children in polygynous marriages compared to monogamous marriages contradicted assertions of many studies referring to the polygyny-fertility hypothesis that fertility was higher in monogamous marriage compared to a polygynous marriage [21, 33]. The study by Turnwait and Alfred (2018) suggested that competition between wives in a polygynous marriage could lead to increase in fertility desire [25]. In Nigeria, women are expected to cooperate with their husbands and recognise their views as mark of respect and obedience. They are not expected to assert their views on family matters but that of their husbands. These factors may help to understand the variance with the polygyny-fertility hypothesis. Mulder (1989) in his study among Kipsigis women in South Western Kenya also found no association between marital status and reproductive performance [24].

The study also indicated that residence in North East and North West regions were related to fertility desire of couples. The Northern part of Nigeria had been noted for high fertility desire and this phenomenon may be associated with Islamic religion and early marriage which encourages polygyny and high fertility, respectively. Fertility intention was found to be associated with the use of contraceptives among couples. The desire for children was higher among couples who were not using contraceptives [22]. Many couples (especially men) would discourage the use of contraceptives due to their high desire for more children, which is often related to religious and cultural beliefs.

### Strengths and limitations of the study

The major strength of this study is the use of 2018 nationally representative Nigeria Demographic and Health Survey datasets collected through a consistent methodology. This makes the findings of the study generalizable. However, this analysis has some drawbacks. Prominently, the analysis utilized cross-sectional data; hence, only associations and no causal relationships are established. Since the survey was self-reported, there are the possibilities of recall and social acceptability biases. Another limitation of the study is that fertility desires of husbands as reported by wives were used for analysis because a fertility report of the husbands was not available. Also, reports of husbands on children ever born were used due to the polygynous nature of many families and the fact that a woman might report her own children ever born rather than that of the family. In addition, it could be that couples were in a polygynous relationship to have more children. There could be some limitations as regards to the accuracy and quality of educational level of respondents, for example, on the part of respondents with little or no formal education.

## Conclusion

The study revealed the relevance of family type to fertility desire among couples in Nigeria. The fertility desire of husbands in a polygynous marriage was about four children compared to fertility desire of one child of husbands in a monogamous marriage. The currently high and stalled fertility level in Nigeria necessitates new approaches to influence fertility desire. Family planning policy and intervention programmes should also target the polygynous family type, especially with focus on men within this family setting due to their high desire for more children. In addition, because of the roles of men as the heads of most families and major decision-makers in the Nigerian society, they should be integrated into such programmes. Predisposing factors of fertility desire for more children, such as the region, religion and contraceptive use of couples, should be addressed in activities towards affecting fertility desire for more children. In addition to awareness campaigns, encouraging couples to use contraceptives with consideration of their religious beliefs should be considered. Therefore, to achieve the Sustainable Development Goals one, three, four and five, existing intervention programmes and policies need evaluation and revision to take cognisance of factors associated with fertility desire for more children among couples.

## Data Availability

Data for this study were sourced from the 2018 Nigeria Demographic and Health Survey (NDHS), available here: https://dhsprogram.com/what-we-do/survey/survey-display-438.cfm.

## Abbreviations

DHS: Demographic and Health Survey
EAS: Enumeration Areas
FOS: Federal Office of Statistics
IRB: Institutional Review Board
NDHS: Nigeria Demographic and Health Survey
NPopC: National Population Commission
SDGs: Sustainable Development Goals
